# Case Report: A Case Series of Rare High-Type Anorectal Malformations With Perineal Fistula: Beware of Urethral Involvement

**DOI:** 10.3389/fsurg.2021.693587

**Published:** 2021-07-15

**Authors:** Lim Hui Jun, Anette Jacobsen, Rambha Rai

**Affiliations:** Department of Paediatric Surgery, KK Women's and Children's Hospital, Singapore, Singapore

**Keywords:** anorectal malformation, pediatric surgery, posterior sagittal anoplasty, urethral involvement, reconstruction

## Abstract

Anorectal malformations (ARMs) are one of the more common congenital anomalies encountered in pediatric surgery where the majority are diagnosed in the early neonatal period. The etiology of ARM remains uncertain and is likely to be multifactorial. A majority of ARMs result from abnormal development of the urorectal septum in early fetal life. There can be a broad range of presentation features varying from low anomalies with perineal fistula to high anomalies mandating intricate management. To develop a standardized system for comparison in follow-up studies, the Krickenbeck classification was introduced according to the type of fistula. According to the Krickenbeck classification of ARM, those with a rectoperineal fistula are classified as low-type ARM and are usually managed with a perineal anoplasty without colostomy. In this case series, we describe two rare cases of distinct high and intermediate ARM with rectoperineal fistulas, which were thought to be low-type ARM but were subsequently found to have urethral involvement. Our cases consisted of high and intermediate ARMs, which were successfully treated with posterior sagittal anorectoplasty as described. These cases exemplified rare variants of ARM where rectoperineal fistulas can be associated with high-type anomalies. Rare-variant ARM with rectopenile or rectoscrotal fistula can be associated with high-type anomalies in contrast to classical rectoperineal fistulas. A high index of suspicion should remain in cases with previous urinary tract infection despite normal imaging. Careful planning is also needed with consideration of possible need for urethral repair during anoplasty, which was needed in both our cases.

## Introduction

Anorectal malformations (ARMs) are among the more commonly seen congenital defects seen in pediatric urological surgery having an incidence from 1 in 2,000–5,000 live births (1). It is more common among Asians, as well as males compared to females (2). This abnormality comprises a spectrum in severity defined by an absent anal opening where the rectum may either communicate with the urinary tract by a fistula or end blind (3). There can be a broad range of presentation features varying from low malformation with perineal fistula requiring simple management to high anomalies requiring intricate management (4). Antenatal diagnosis of an isolated ARM is uncommon where the majority of cases are diagnosed in the early neonatal period (5). Most of the patients present within the early neonatal period with obstructive symptoms attributed to an absent or narrow fistula (6). Furthermore, ARM may be associated with congenital anomalies, which have been described in 50% of cases, including cardiac and vertebral defects as well as chromosomal abrnormalities (7). According to the Krickenbeck classification of ARM, those with a rectoperineal fistula are classified as low-type ARM and are usually managed with a perineal anoplasty without colostomy. In this case series, we describe two rare cases of distinct high and intermediate ARM with perineal fistula in males successfully treated with surgery. Notably, there are only a few case reports of ARMs with perineal fistula of the rare high-type variant (8–10).

## Ethics Statement

Written informed consent was obtained from minor's legal guardian for the publication of any potentially identifiable images or data included in this article.

## Case Report

**Figure 1:**
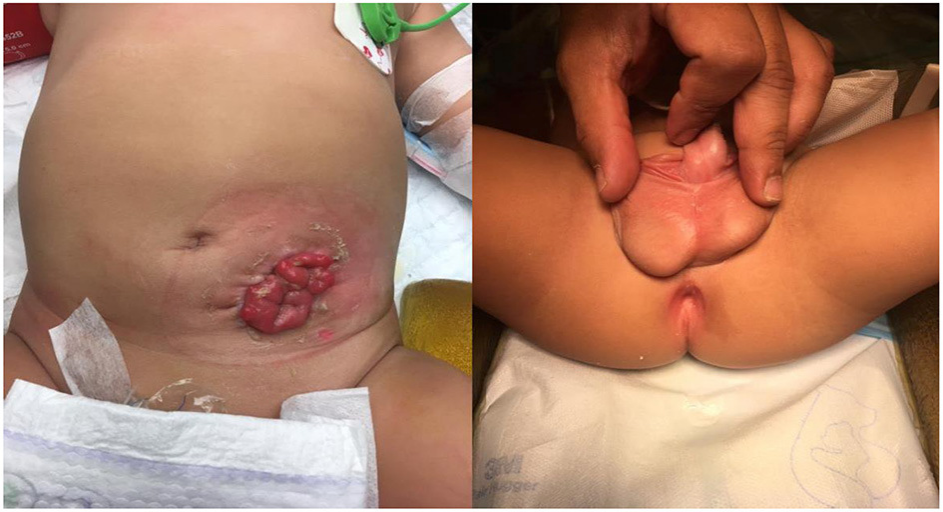
Preoperative photographs of the intermediate anorectal malformation with rectoperineal fistula.

**Figure 2:**
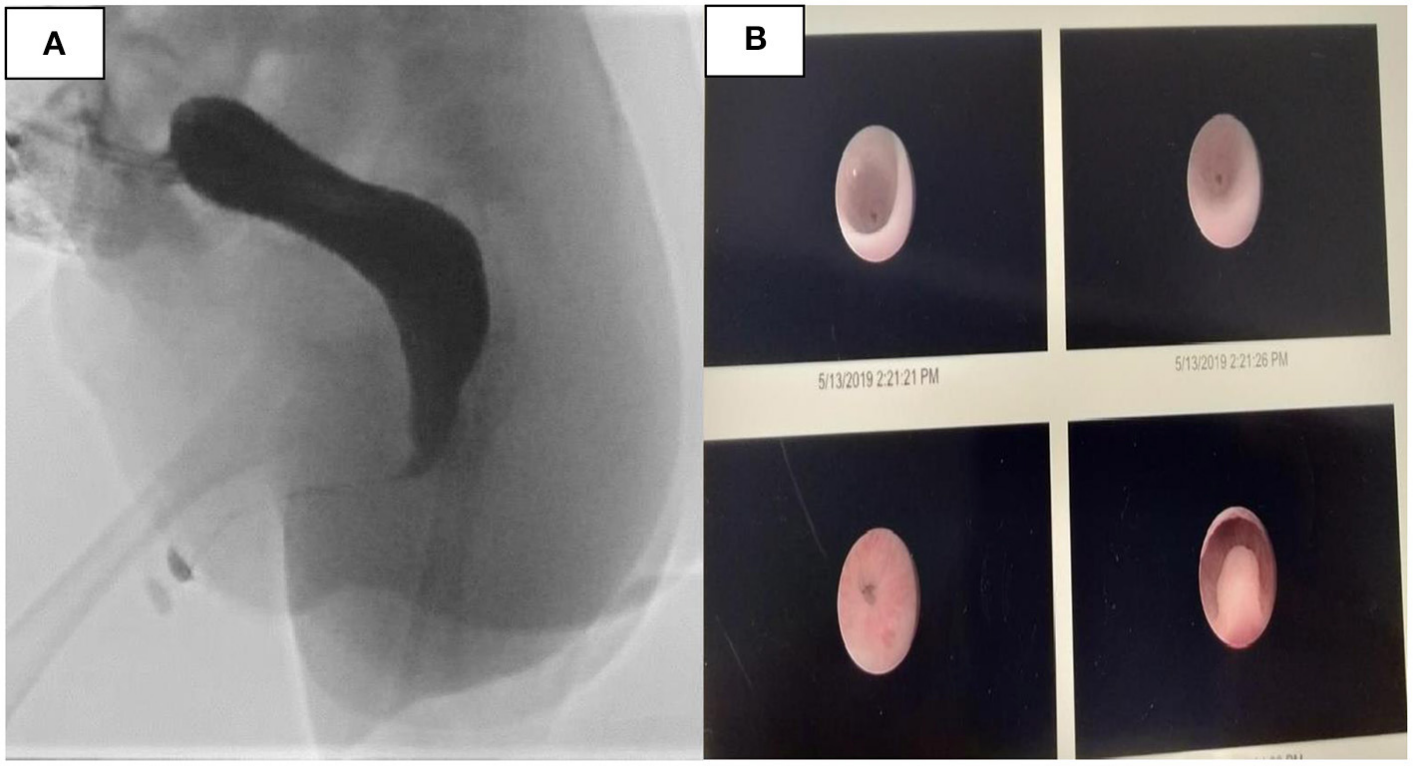
**(A)** Distal loopogram showed a rectocutaneous fistula located at the anterior perineum. **(B)** Cystoscopy showed no obvious internal fistula with good stream on bladder compression. There was a reddish dot at 6-o'clock position on the posterior urethra.

**Figure 3:**
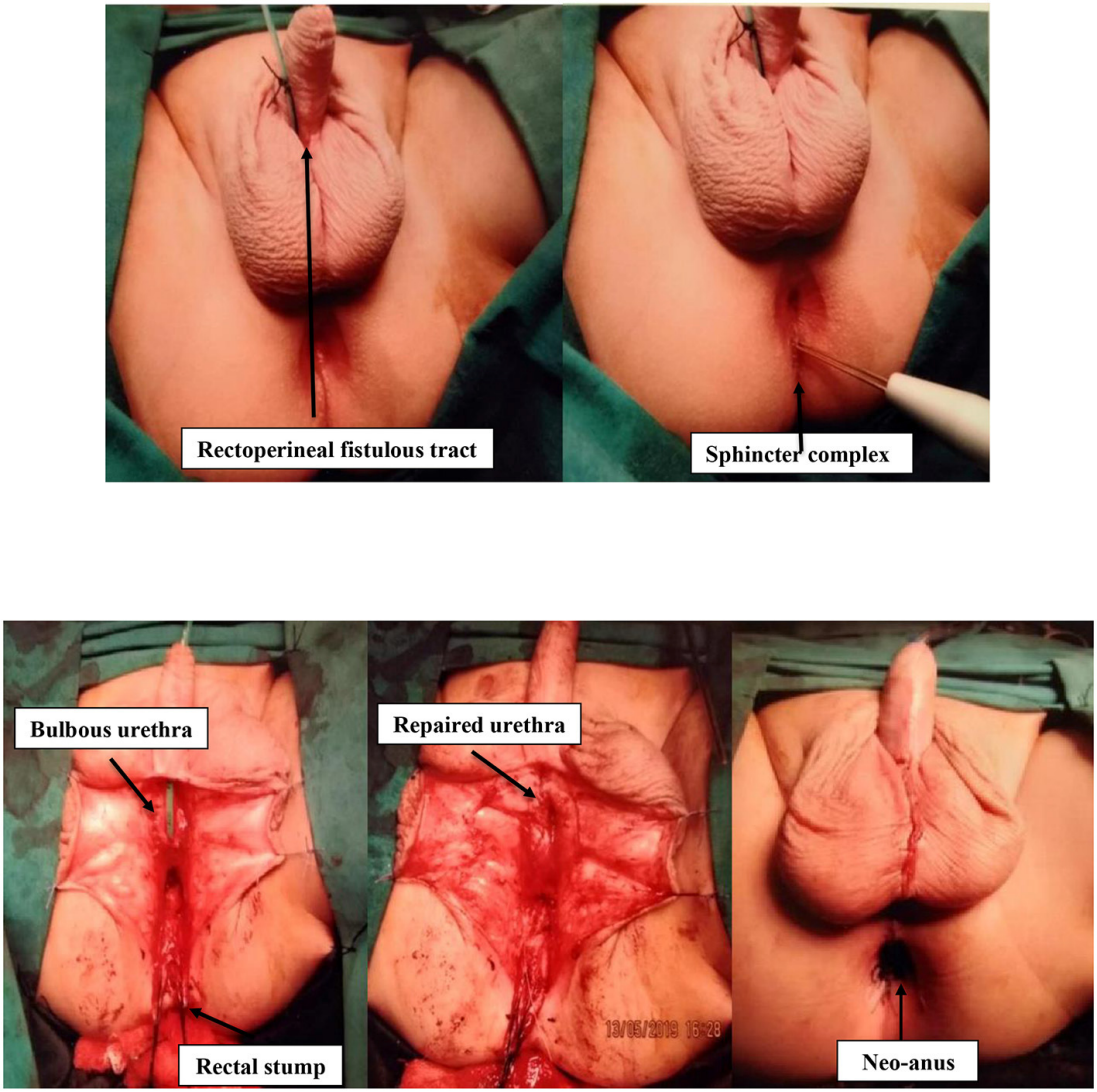
Intraoperative photographs demonstrating that a 1-mm rectoperineal fistula was noted externally and cannulated with a microprobe. On mobilization of the rectum, the rectal stump was found to be at the level of the bulbous urethra. A common wall was identified between the bulbous urethra and the fistula tract extending for 3 cm. The bulbous urethra was inadvertently laid open during separation of the common wall. Subsequently, urethroplasty was done in two layers, and anoplasty was completed.

The first case is a 4-months-old male infant with known ARM (Figure 1) who underwent colostomy creation in his native country. Antenatally, he had placental placement issues, which settled at 22 weeks' gestation. There was no oligohydramnios or polyhydramnios. He was born at full term. There was no passage of mucous or meconium per urethra at birth. Notably, he had one previous episode of urinary tract infection. There were no other congenital anomalies. Investigations included a distal loopogram, which showed a rectocutaneous fistula located at the anterior perineum (Figure 2). Ultrasound of the kidneys, bladder, and spine were normal. Micturating urethrogram showed no vesicoureteric reflux or urethral fistula. Cystoscopy showed no internal fistula with good stream on bladder compression. There was a reddish dot at 6-o'clock position at posterior urethra (Figure 2). Subsequently, the patient underwent anorectoplasty and urethral repair (Figure 3). Intraoperative findings included a penoscrotal dimple with 1-mm rectoperineal fistula, which was able to be cannulated with microprobe. Rectal stump was found at perineum at level of bulbous urethra. The fistula appeared small and tapered to connect at the level of the bulbous urethra. The common wall between the bulbous urethra and rectal fistulous tract was ~3 cm. The bulbous urethra, which measured ~3 cm, was laid open inadvertently while separating the common wall. Urethroplasty was done in two layers with Vicryl 6-0 after placing an 8-Fr feeding tube. Postoperatively, recovery was uneventful, and stoma closure was performed 3 months later. The patient is currently 10 months old and voiding per urethra with no further urinary tract infections. He is continent of both bladder and bowel movement with no constipation.

**Figure 4:**
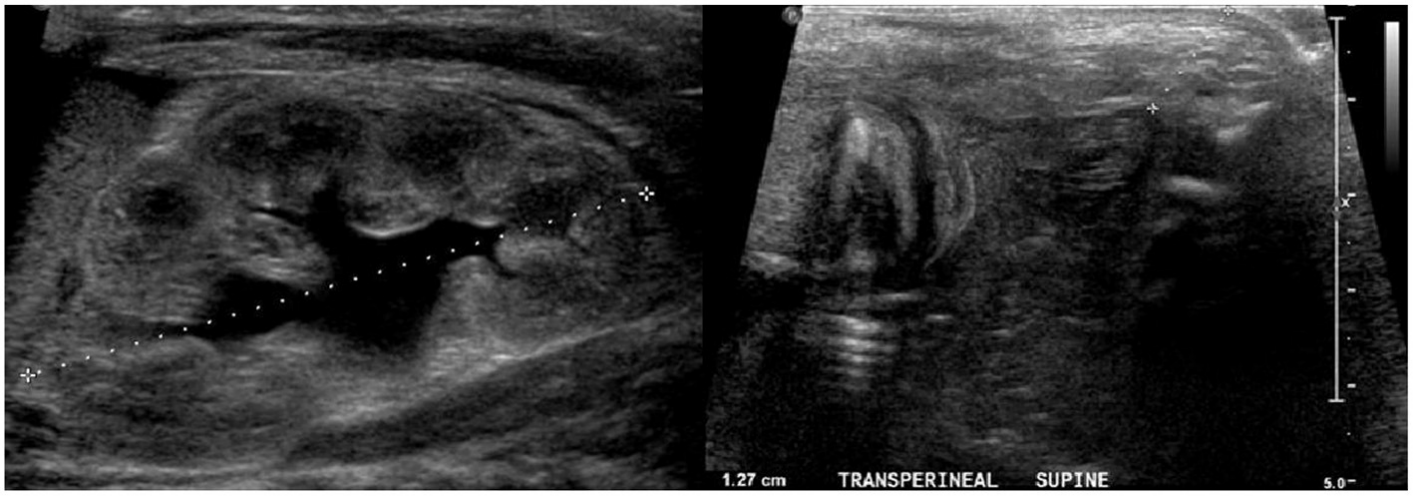
Ultrasound of the kidneys, ureter, bladder, and perineum demonstrated a dilated left pelvicalyceal system. There was a distal rectal pouch to the perineum that measured 12 mm.

**Figure 5:**
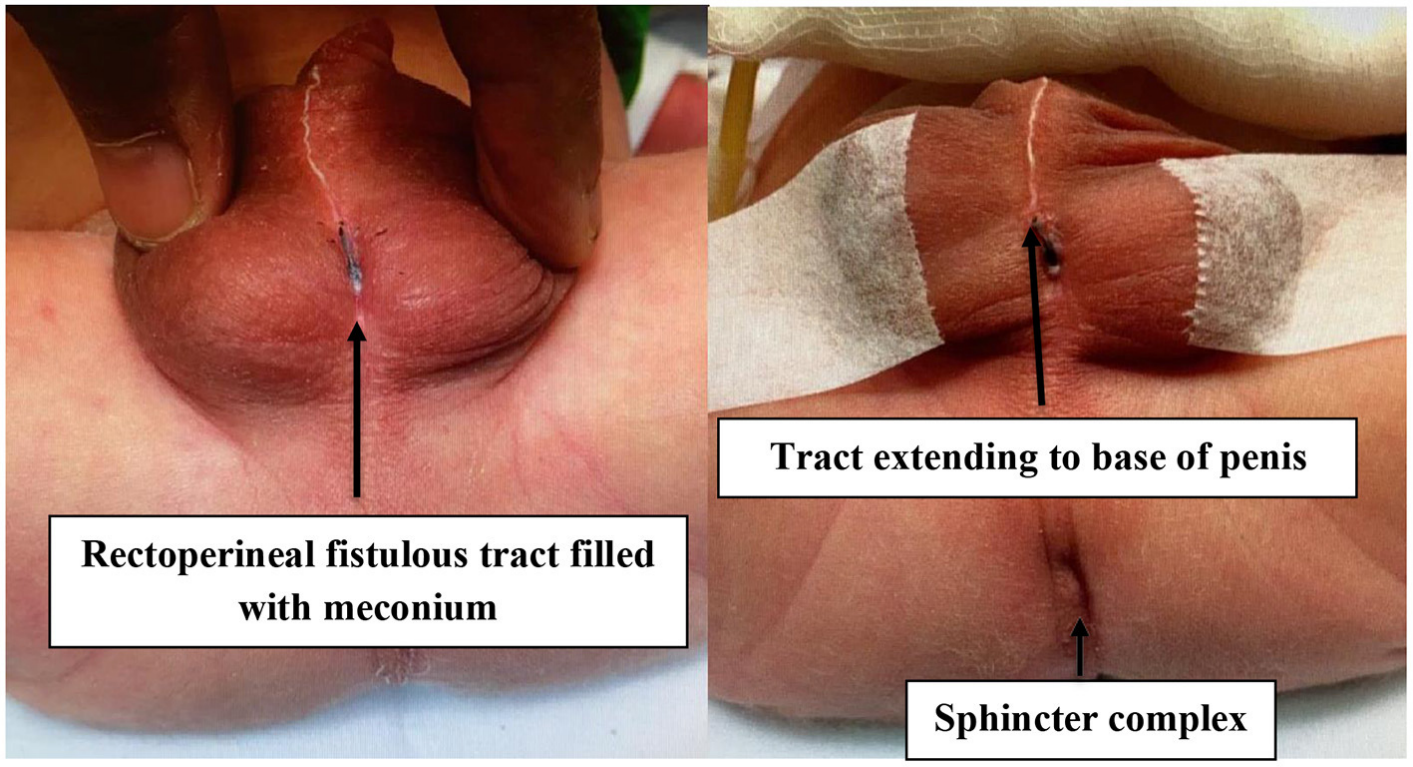
The baby was initially planned for perineal anoplasty on day 2 of life. It was noted there was a fine, long cutaneous track running in the scrotal raphae in close proximity with the bulbar urethra extending all the way to the base of the penis. The bulbar urethra was inadvertently entered during separation of the thin common wall. This was repaired in two layers. Anoplasty was then performed.

The second case is a full-term male infant who was antenatally well with no oligohydramnios or polyhydramnios. He was delivered via normal vaginal delivery. There was no passage of mucous or meconium per urethra at birth. At birth, he was diagnosed with an ARM with rectoperineal fistula opening at scrotal raphe with bifid scrotum appearance. Abdomen x-ray showed rectal gas below the pubococcygeal line consistent with a low imperforate anus. Ultrasound of the kidney, ureters, bladder, and perineum showed a dilated pelvicalyceal system (Figure 4). Distal rectal pouch to the perineum measured 12 mm. Subsequently, he underwent a single-stage anorectoplasty and urethral repair. Intraoperative findings included a high-type ARM with perineal fistula with opening at scrotal raphe creating a bifid scrotum (Figure 5). A fine, long cutaneous track running in the scrotal raphe was in close proximity with the bulbar urethra extending all the way to the base of the penis. The fistula tract was closely adherent to the bulbar urethra, rectum, and supralevator. The bulbar urethra was inadvertently breeched during separation of the thin common wall, which was repaired in two layers. Subsequently, an anoplasty was performed where an incision was made surrounding the fistula and the tract dissected posteriorly until the site of the anus. The external sphincter fibers were accurately mapped using a nerve stimulator, and a posterior midline incision was made in the rectal pouch. The mucosa and anoderm at the cut edges were then approximated with absorbable sutures. Postoperatively, recovery was uneventful, and the patient was discharged satisfactorily. Notably, he had a few VACTERL (vertebral defects, anal atresia, cardiac defects, tracheoesophageal fistula, renal anomalies, and limb abnormalities) associations, including a sacral defect with spinal lipoma for which he underwent laminectomy at 9 months old. In addition, he had an atrial septal defect with patent ovale, which was conservatively managed following review by the cardiothoracic surgeon. He is currently 6 years old and is thriving with good weight gain. He is voiding normally per urethra with no incontinence or urinary tract infection. He has mild constipation on low-dose laxatives.

## Discussion

ARMs have been reported from 1 in 2,000 to 5,000 live births, with an inclination toward males (1). They demonstrate a range of abnormalities that have been reportedly derived from deficient partition of the cloaca and maldevelopment of the perineal mound and genital folds (11). In male patients, the quick and extensive growth of the perineum usually prevents fistulas from developing at the front of the scrotum, defining low-type anomalies in males, which can present with an enteroperineal or enterourinary tract fistula (11). The origin of these malformations remains uncertain and is likely attributed to multiple factors (12, 13). Male patients with ARM typically present within the initial 4 or 5 days of life as seen in our cases (14, 15). The absence of the symptoms despite a high type of ARM is due to the broad fistulous communication between the rectum and the urinary tract (16–18). There appears to be a low rate of inheritance in family members; however, some appear to have an autosomal dominant inheritance pattern (19).

The type of ARM is essential for classification and guides the recommended treatment plan. The initial classification of ARM was based on the position of the rectum in comparison to the levator ani or pelvic floor. Wingspread classification was established according to the level of the arrest of rectal descent and patient's gender (20). However, after the introduction of the posterior sagittal anorectoplasty (PSARP) approach by Peña (6), the site of the fistula had important implications in the treatment approach of these patients. Furthermore, improvements in the imaging techniques with greater in-depth understanding of the embryology, anatomy, and pathophysiology of ARM cases have aided the diagnosis and classification of this condition. The new classification system was proposed in 1995, which was grouped according to gender and based on the presence and position of the fistula arising from their experience with PSARP (21, 22). Although PSARP soon became the gold-standard surgery globally, the outcomes of studies assessing long-term result between PSARP and other classical operations were highly variable because of differences in the follow-up criteria. To standardize the methodology for evaluation of patient outcomes, the Krickenbeck group developed a classification that incorporated criteria from both Wingspread and Peña (23). It comprised three components, namely, a diagnostic category, a surgical procedure category, and a category documenting functional outcome criteria. ARMs are termed as high, intermediate, or low according to the position of the blind rectal pouch in relation to the muscle complex of the pelvic floor. There is a range of malformation ranging from a distal atresia of the anal canal to a high rectal atresia (23). These have been termed as high or low, depending on the relationship of the rectum with the pelvic floor and anal sphincter (23). A fistula typically connects the atretic bowel to perineal skin or urogenital tract (24). The exact anatomic configuration corresponds with the level of the atretic malformation, where the higher lesions tend to open anterior to the perineal body. Specifically, a distance larger than 1 cm from the perineum to the rectal pouch in a full-term infant is classified as a high lesion demonstrated in our second case (24). The presence of a fistulous opening in the perineum in the region of the anal pit accompanied by a formed anal dimple and a well-formed midline bottom cleft is usually regarded as evidence of a low ARM. Notably, position of the fistula is used to determine operative management. Specifically, an anoperineal fistula exiting between the scrotum and the anal dimple is usually present in low cases, which can be treated by PSARP, whereas a rectourethral or rectovescical fistula accompanies a high-type ARM, where a diverting colostomy is generally performed first (25). The Krickenbeck classification was utilized in our case reports. More refined imaging modalities and advances in radiological techniques have also made it possible to exactly delineate the site of rectourethral fistula (26). Notably, our cases represent a rare and unique presentation of a high and intermediate ARM associated with a perineal fistula, which are typically linked with a low ARM instead. Perineal fistula associated with a high ARM has been described previously in a few case reports but does not appear within the standard classification system. As such, this highlights the importance of delineating the abnormality prior to intervention. Prior to the definitive surgery, a pressured distal colonogram and voiding cystourethrogram should be performed to reveal the site of rectourethral fistulas. Overall, our cases consisted of a high and intermediate ARM, which were successfully treated with PSARP as described. These cases exemplified rare variants of ARM where rectoperineal fistula can be associated with high-type anomalies. Rare-variant ARM with rectopenile or rectoscrotal fistula can be associated with high-type anomalies in contrary to classical rectoperineal fistulae. Management of these cases should be planned carefully, with possible need of urethral repair during anorectoplasty. Hence, a high index of suspicion should remain in cases with previous urinary tract infection despite normal imaging. Careful planning is also needed with consideration of a possible need for urethral repair during anoplasty, which was needed in both our cases.

There are only a few case reports of ARM with perineal fistula of the rare high-type variant (8–10). For example, a case report from the United Kingdom described a full-term male infant who presented with marked abdominal distension and vomiting at 48 h of life with meconium in the urine (8). He had an imperforate anus with a median skinfold within a visible pit in the perineal skin. Electrical stimulation showed appropriate sphincter contraction surrounding the anal pit. Subsequently, limited exploration of the perineum was attempted, and it was not possible to identify an atretic anal canal or the fistula; hence, a sigmoid colostomy was created. At 3 months of age, a contrast study showed a narrow 2-cm fistula from a high-ending rectum to the anal pit. Thereafter, successful anorectal reconstruction was performed using PSARP. Likewise, a case report from India presented a 9-month-old male infant with imperforate anus who underwent a loop colostomy at 2 days of age (9). There was no fistula noted at the time colostomy creation. With the likelihood of a low ARM, a distal colostogram was done, which demonstrated a faint opacification of the fistula, which was communicating with the opening located over the ventral aspect of the penis and a very low-placed anorectum. A limited PSARP was performed where the possibility of a low ARM with an anocutaneous fistula was high. On exploration, the blind anorectum was demonstrated to be supralevator. As such, a formal PSARP was then performed with ligation of the rectopenile fistula. Similar to our cases, these case reports highlighted the need for caution when a perineal fistula is identified in a flat-bottom or sacral anomaly, which is usually associated with a high lesion and when a fistula tract is unable to be cannulated. These features suggest the possibility of a rectoperineal fistula rather than an anocutaneous fistula. These rare anomalies should be highlighted when a fistula is seen in the perineum, and a diverting colostomy is recommended as the initial surgical procedure prior to definitive operation.

The management of ARM has evolved from classical procedures to PSARP and even minimal invasive approaches with laparoscopic abdominoperineal rectoplasty techniques (27). Together with these refinements, there has been a paradigm shift in approach to these patients, which involves holistic approach to management of ARM with a goal to achieve complete fecal and urinary continence in conjunction with a favorable quality of life. In terms of the long-term function outcome of males with ARM, it carries a favorable outlook where a study reported a low 15% impaired urinary and bowel function outcome (28). In these cases, intermittent soiling and constipation have been associated with neurological damage and developmental delay, insufficient long-term follow-up and care, and presence of a tethered cord. Moving forward, it would be insightful to assess the long-term outcome of bowel continence for our patients who have undergone posterior sagittal anorectoplasty, which potentially has a significant impact on quality of life. Furthermore, useful scoring systems to evaluate the outcome of ARM repair could be incorporated into clinical practice. These include the Kelly's and Kiesewetter-Chang scoring systems (29, 30).

## Conclusion

ARMs are uncommon but complex congenital anomalies that require an individualized treatment strategy. These treatment strategies are unique to each patient and essential to improve functional outcomes. Rare-variant ARM with rectopenile or rectoscrotal fistula can be associated with high-type anomalies in contrary to classical rectoperineal fistula. These rare anomalies should be highlighted when a fistula is seen in the perineum, and a diverting colostomy is recommended as the initial surgical procedure prior to definitive operation. Atypical presentations may not necessarily follow conventional treatment approaches, and a high index of suspicion should remain in cases with previous urinary tract infection despite normal imaging. Careful planning is also needed with consideration of a possible need for urethral repair during anoplasty, which was needed in both our cases.

## Data Availability Statement

The raw data supporting the conclusions of this article will be made available by the authors, without undue reservation.

## Ethics Statement

Written informed consent was obtained from minor's legal guardian for the publication of any potentially identifiable images or data included in this article.

## Author Contributions

All authors listed have made a substantial, direct and intellectual contribution to the work, and approved it for publication.

## Conflict of Interest

The authors declare that the research was conducted in the absence of any commercial or financial relationships that could be construed as a potential conflict of interest.
